# Galectin-1 is overexpressed in CD133^+^ human lung adenocarcinoma cells and promotes their growth and invasiveness

**DOI:** 10.18632/oncotarget.3076

**Published:** 2014-12-26

**Authors:** Xuefeng Zhou, Dan Li, Xianguo Wang, Bo Zhang, Hua Zhu, Jinping Zhao

**Affiliations:** ^1^ Department of Thoracic and Cardiovascular Surgery, Zhongnan Hospital, Wuhan University, Wuhan, China; ^2^ Department of Surgery, Davis Heart and Lung Research Institute, The Ohio State University Wexner Medical Center, Columbus, Ohio, USA

**Keywords:** galectin-1, CD133, lung adenocarcinoma, Cancer Stem Cells

## Abstract

Previous studies demonstrated that a subpopulation of cancer cells, which are CD133 positive (CD133^+^) feature higher invasive and metastatic abilities, are called cancer stem cells (CSCs). By using tumor cells derived from patients with lung adenocarcinoma, we found that galectin-1 is highly overexpressed in the CD133^+^ cancer cells as compared to the normal cancer cells (CD133^−^) from the same patients. We overexpressed galectin-1 in CD133^−^ cancer cells and downregulated it in CSCs. We found that overexpression of galectin-1 promoted invasiveness of CD133^−^ cells, while knockdown of galectin-1 suppressed proliferation, colony formation and invasiveness of CSCs. Furthermore, tumor growth was significantly inhibited in CSCs xenografts with knockdown of galectin-1 as compared to CSCs treated with scramble siRNAs. Biochemical studies revealed that galectin-1 knockdown led to the suppression of COX-2/PGE2 and AKT/mTOR pathways, indicating galectin-1 might control the phenotypes of CSCs by regulating these signaling pathways. Finally, a retrospective study revealed that galectin-1 levels in blood circulation negatively correlates with overall survival and positively correlates with lymph node metastasis of the patients. Taken together, these findings suggested that galectin-1 plays a major role on the tumorigenesis and invasiveness of CD133^+^ cancer cells and might serve as a potential therapeutic target for treatment of human patients with lung adenocarcinoma.

## INTRODUCTION

Lung cancer is the leading cause of cancer-related mortality and the second most common cancer for both men and women worldwide [1]. The adenocarcinoma is the most common lung cancer and accounts for almost half of all non-small cell lung cancers (NSCLC), which occupied 85% of total lung cancers [2, 3]. Despite continuous efforts devoted to improving the therapeutic response (such as gefitinib and erlotinib combinative chemotherapy) [4], the overall five-year survival rate of advanced NSCLC is still less than 15% [5, 6]. Therefore, the identification of novel therapeutic targets is of particular importance for the treatments of lung cancers.

In the last decade, accumulating evidence has supported the notion that the tumors contain a minor subpopulation, which are responsible for the tumorigenesis, termed as cancer stem cells (CSCs) based on the fact that they share some features with the normal stem cells such as self-renewal, multiple differentiation and tumor initiation abilities [7, 8]. The same concept is also applicable to lung cancers [9, 10]. To date, CD133 has been recognized as a stem cell surface marker, and is being currently used for identification or isolation of the putative cancer stem cell population from malignant tumors [9, 11, 12]. A population of CD133^+^ stem-like cells has also been observed in lung cancers and demonstrated that this cell subpopulation is capable of generating unlimited progeny of differentiated cells that constitute the tumor mass [9, 13, 14]. Furthermore, CD133-expressing tumor cells has been found to mediate chemo-/radio-therapy resistance [15] and metastasis in lung cancers [16], thus, molecular and functional characterization of such population may provide valuable information to development of effective therapies for treatment of lung cancers.

Galectin-1 (gal-1), a member of the β-galactoside-binding protein family, is a multifunctional protein capable of regulating multiple processes such as tumor cell adhesion, proliferation, differentiation, apoptosis, invasiveness and metastasis [17-20]. Acting both intracellarly and extracellularly, gal-1 has been shown to promote cancer progression, chemoresistence, and induce dendritic cells anergy through upregulating COX-2/PGE2 pathway in lung cancers [21, 22]; furthermore, it can also stimulate embryonic stem cell proliferation through activation of Akt/mTOR signaling pathway [23], which plays a central role in regulating cell growth, proliferation, and survival. The dysregulation of Akt/mTOR pathway was reported to contribute to lung cancer development and maintenance [24, 25]. Thus, we hypothesized that gal-1 might be involved in tumorigenesis and invasiveness of CSCs in lung adenocarcinomas.

In the present study, we first investigated the expression of gal-1 in CD133^+^ cancer stem cells derived from human patients with lung adenocarcinoma. We found that gal-1 was overexpressed in CD133^+^ tumor cells when compared with CD133^−^ cells from the same tumor tissue in nine patients with lung adenocarcinoma. In order to examine the function of gal-1, we performed *in vitro* experiments to knockdown galectin-1 in CD133^+^ tumor cells, we found that this treatment significantly reduced tumor cells proliferation, colony formation and invasion. In contrast, when we overexpressed gal-1 in CD133^−^ tumor cells, although gal-1 overexpression does not alter proliferation of these cells, it significantly promotes their invasive ability as demonstrated by matrigel invasion assay. Consistent with cell culture studies, our *in vivo* xenograft assay also revealed that knockdown of gal-1 led to suppression of tumor growth in SCID mice. Molecular analysis suggested that the tumor suppression effects of gal-1 silencing might be due to downregulation of COX-2/PGE2 and AKT/mTOR pathways. Finally, we found that high level of gal-1 in blood circulation is closely correlated with a poor prognosis and high lymph node metastasis of the patients with lung adenocarcinoma. Therefore, our data suggested an important role of gal-1 in CD133^+^ lung cancer stem cells, which might be a potential target for treatment of lung adenocarcinoma and a marker for prognosis of lung cancer patients.

## RESULTS

### Serum galectin-1 level and CD133^+^ cancer cells are positively correlated with the disease progression of the patients with lung adenocarcinomas

**Figure 1:**
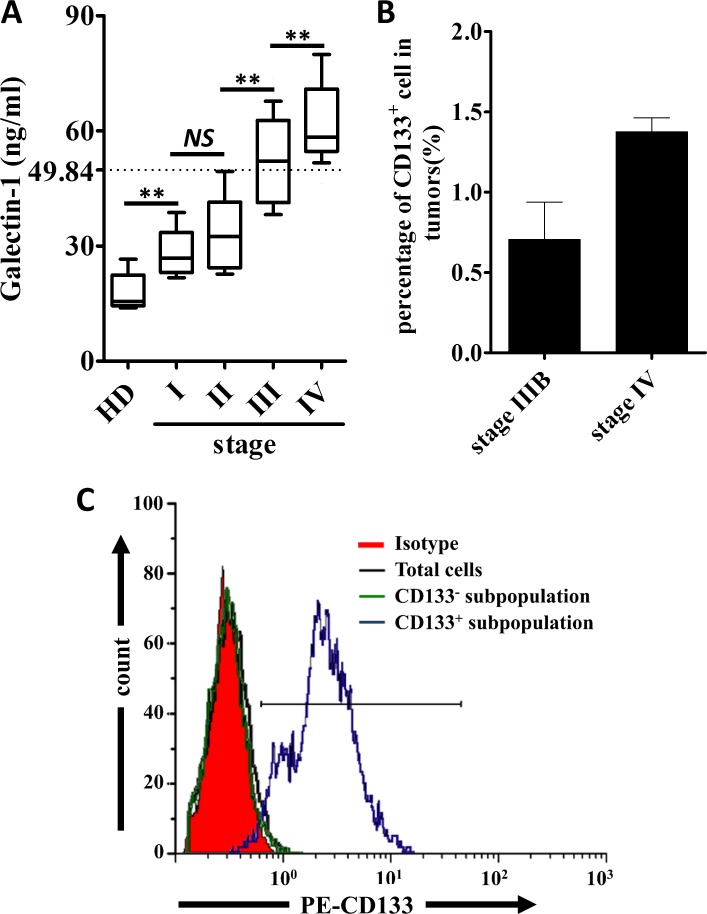
Serum level of Galectin-1 and percentages of CD133 cells are positively correlated to the stage of the human patients with lung adenocarcinoma (A) Serum levels of galectin-1 in health donors (HD) and patients with different stages of lung adenocarcinoma were detected by ELISA (n=75, serum collected from enrolled patients or tumor serum bank). Spearman rank test was performed to show a positive correlation between cancer stage and serum galectin-1 level. (p=7.3e-18, R=0.8) (B) The percentages of CD133^+^ cells in tumor tissues from different stages of lung cancer patients were analyzed by flowcytometry (n=2 patients at stage IIIB and n=7 patients at stage IV). (C) CD133^+^ cells in tumor tissues were sorted by flowcytometry, and then the purity of sorted cells was confirmed. To show the enrichment of CD133^+^ cells, total tumor cells and CD133^−^ subpopulation were used as controls. Data presented as Mean ± SD (n = 3 independent experiments, ** p<0.01).

The demographic and clinical features of sixty six patients at different stages of lung carcinomas are summarized in Table 1. We first examined the serum level of gal-1 in 66 patients and health donors. Compared with health donors, gal-1 level was significantly higher in patients with lung adenocarcinoma (Fig. 1A). More importantly, the serum gal-1 level positively corrects with the stage of the patients (p=7.3e-18, R=0.8; Spearman rank test) indicating gal-1 might play a role in progression of lung adenocarcinoma. We next examined the percentage of CD133^+^ cancer cells in the whole cancer cells population. We found that there was a trend that higher percentages of CD133^+^ cells were presented in stage IV lung adenocarcinomas (n=7 patients) as compared to those in stage IIIB cancers (n=2 patients) (Fig. 1B). Combining data from Fig. 1A and Fig. 1B, we can draw a positive correlation between serum level of gal-1 and presence of CD133^+^ cells in human patients. Thus, we hypothesized that CD133^+^ cells might overexpress gal-1 and contribute the high level of gal-1 in blood circulation. In order to determine the differences between CD133^+^ and CD133^−^ cancer cells in production of gal-1, we established a magnetic cell separation protocol to purify CD133^+^ cells from the whole tumor cell population for our following studies. The purity of CD133^+^ cancer cells isolated from biopsies of human patients with lung carcinoma was then analyzed by flow cytometry. As shown in Fig. 1C, flow cytometry data indicated a good enrichment of CD133^+^ subpopulations by our meganetic cell separation protocol, thus, we followed this protocol to isolate the cells for the following studies.

**Table 1:** Clinicopathologic features of pulmonary adenocarcinoma patients

Characteristic	cases(%)	specimens†		Gal-1 (ng/ml)*	P value
		Yes	No		
Total	66	9	57	48.65 ± 19.28	
Sex					0.1377
Male	44(66.7%)	5	39	50.99 ± 19.27	
Female	22(33.3%)	4	18	43.96 ± 18.80	
Median age (years. range)	57.3 (38-77)	54.6 (42-76)	58.9 (38-77)		
Smoking status					0.0085
Current or former smoker	38(57.6%)	7	31	52.73 ± 18.08	
Non-smoker	28(42.4%)	2	26	40.48 ± 19.34	
TNM stage					<0.0001
I	7(10.6%)	0	7	28.71 ± 6.184	
II	8(12.1%)	0	8	33.30 ± 9.615	
III	19(28.8%)	2	17	52.67 ± 11.04	
IV	32(48.5%)	7	25	63.04 ± 11.75	
Lymphnode metatasis					<0.0001
Yes	56(84.8%)	9	47	56.61 ± 14.68	
No	10(15.2%)	0	10	31.54 ± 8.322	

† Yes: patients with tumor tissues and serum samples; No: patients with only serum samples.

* Mean ± SD.

### Galectin-1 is upregulated in CD133^+^ lung adenocarcinoma cells

**Figure 2:**
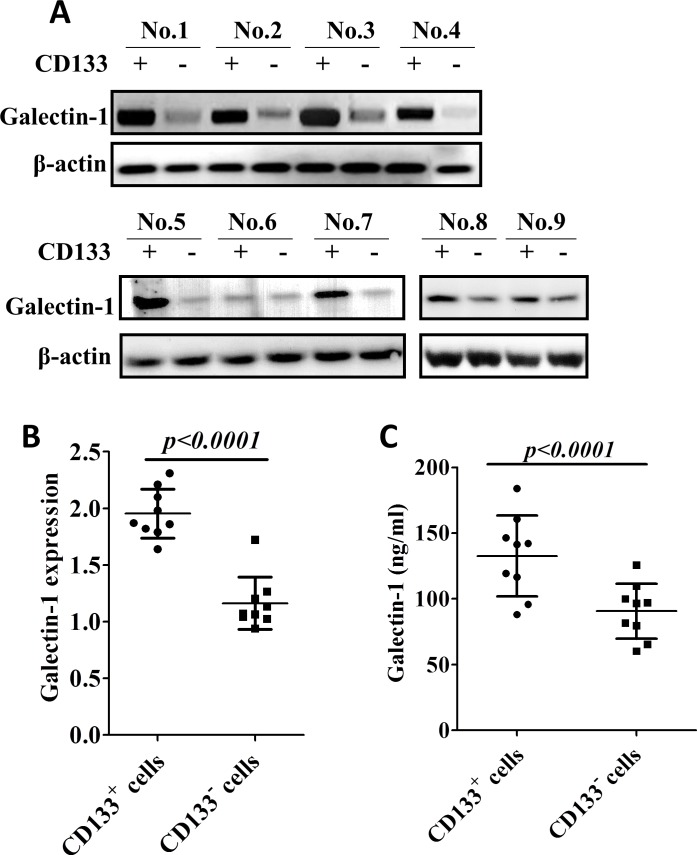
Galectin-1 is overexpressed in CD133 human lung adenocarcinoma cells (A) Tumor biopsies from 9 patients were disassociated and sorted by CD133 expression. The expression of galectin-1 in the cell lysates of CD133^+^ and CD133^−^ subpopulations were subjected to Western blot analysis. The results show galectin-1 is overexpressed in CD133^+^ subpopulation as compared to that in CD133^−^ subpopulation. (B) mRNA levels of galectin-1 in two subpopulations were determined by real time RT-PCR. Expression of β-actin was used as internal control. (C) CD133^+^ and CD133^−^ cells were cultured separately. Galectin-1 levels in media of two subpopulations were determined by ELISA. Data presented as Mean ± SD, n = 3 independent experiments.

To examine the expression of gal-1 in two subpopulations of lung adenocarcinoma cells, we performed Western blotting by using CD133^+^ and CD133^−^ cancer cell lysates from different human patients, as shown in Fig. 2A, CD133^+^ cells express significantly higher gal-1 than CD133^−^ cells from the same patient (gal-1 is highly upregulated in CD133^+^ cells in eight out of nine patients, while CD133^+^ cells derived from patient No. 6 only showed minor overexpression of gal-1). Consistent to Western blot results, elevated mRNA levels of gal-1 were found in CD133^+^ cells than that in CD133^−^ cells of the same patient (Fig. 2B). To determine whether the elevation of gal-1 in blood circulation is results of secretion of gal-1 from CD133^+^ cells, we also compared gal-1 concentrations in media from CD133^+^ and CD133^−^ cells cultures. As shown in Fig. 2C, gal-1 in media of CD133^+^ cells was about 1.46 folds higher than that of CD133^−^ cells. In summary, these results indicated that gal-1 was highly expressed in CD133^+^ cells as compared with that in CD133^−^ cells.

### Knockdown of Galectin-1 inhibits the proliferation, Colony forming efficiency and invasion of CD133^+^ cells

**Figure 3:**
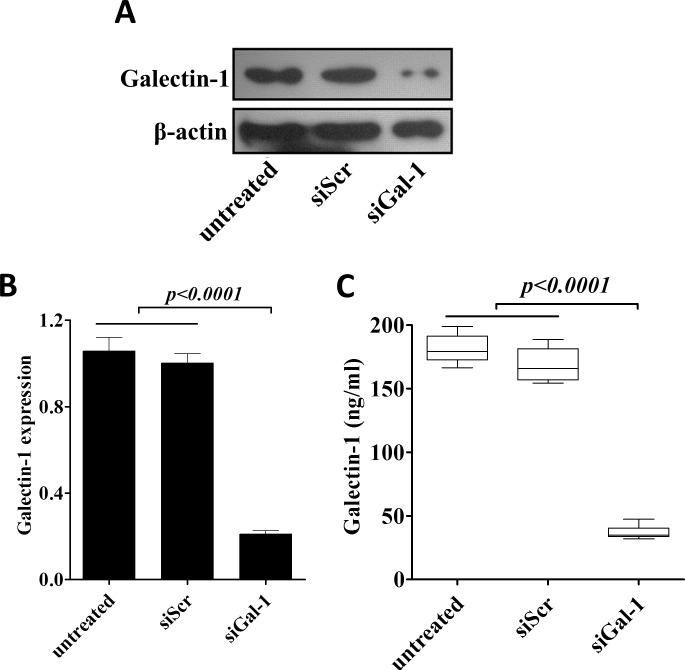
Generation of stable knockdown of galectin-1 CD133^+^ cells siRNA mediated knockdown of gal-1 was confirmed by western blotting (A), real time RT-PCR (B) and presence of gal-1 in culture media (C). Data presented data showed as Mean ± SD, n = 3 independent experiments.

Our next question was whether overexpression of gal-1 contributed to the phenotypes of CD133^+^ cells? To test this possibility, we transfected siRNA specific against gal-1 in CD133^+^ cells, as shown in Fig. 3A and 3B, both protein and mRNA levels of gal-1 were greatly suppressed by siGal-1 treatment as compared to untransfected or scramble siRNA (siScr) transfected cells. In addition, secretion of gal-1 was also significantly suppressed by siGal-1 treatment (Fig. 3C).

**Figure 4:**
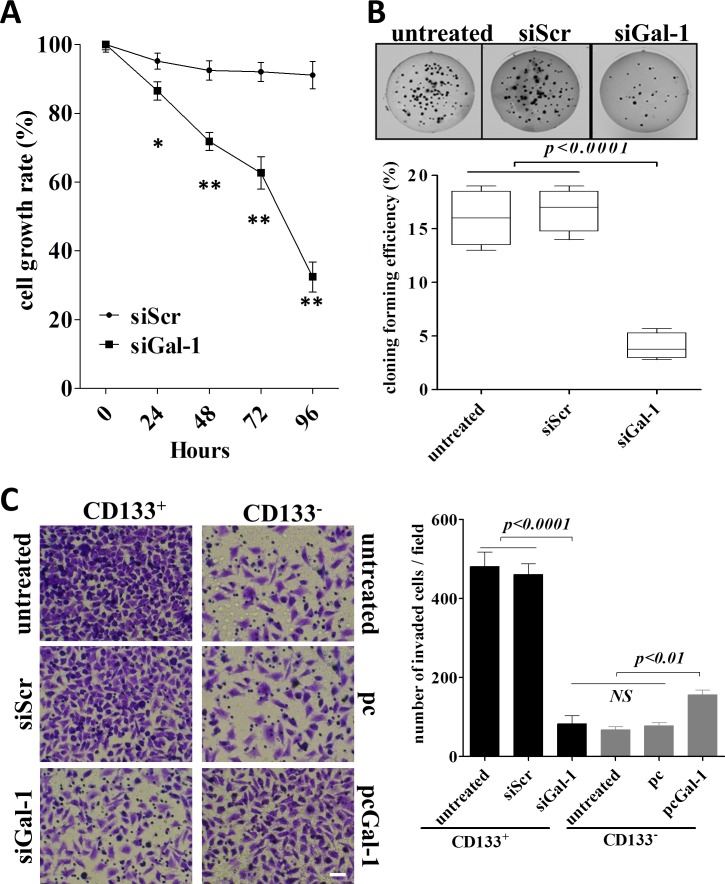
Stable knockdown of galectin-1 in lung adenocarcinoma results in suppression of proliferation, anchorage-independent growth and invasion *in vitro* (A) The proliferation of CD133^+^ cells treated with shRNA against galectin-1, scramble shRNA or untransfected controls were measured by MTT assay. The proliferation rate was determined as absorbance of transfected (either shRNA against galectin-1 or scramble shRNA) CD133^+^cells/absorbance of untransfected CD133^+^cells. (B) CD133^+^ cells treated with different constructs (1×10^3^ cells per condition) were seeded in soft agar for evaluating anchorage-independent tumor growth. Representative images show staining of colonies in each treatment (*upper panels*). The colony formation efficiencies of indicated treatments were summarized (*lower panel*). (C) CD133^+^ or CD133^−^ cells (5×10^4^ per group) in 0.5% FBS-DMEM were plated in the upper chamber coated with Matrigel, and 10% FBS-DMEM was added to the lower chamber. After 48 h incubation, the cells invaded to the outer surface of the the upper chambers were stained and counted. Six random fields were photographed (shown in *left panels*) and summarized (*right panel*) per upper chamber in each group. Data presented as Mean ± SD, n = 3 independent experiments. * p<0.05, ** p<0.01. The scale bar: 50 μm.

To test the effects of siGal-1 on the behaviors of CD133^+^ cells, we performed experiments to determine the proliferation, colony formation and invasion of the cells. We found that SiGal-1 treated cells displayed greatly reduced proliferation (Fig. 4A), anchorage-independent colony growth (Fig. 4B) and invasion (Fig. 4C) as compared to the untransfected cells or the cells treated with siScr. Therefore, our data indicated that gal-1 is required for the phenotypes of CD133^+^ cells. In particular, the ability of invasion is a character of CD133^+^ lung cancer stem cells, which possess the capacity to migration and invade to form distant metastases [26, 27].

If gal-1 is important for the phenotypes of cancer stem cells, one would expect that overexpression of gal-1 in CD133^−^ cells would promote the cells to be more aggressive as CD133^+^ cells. Indeed, as shown in Fig. 4C, control CD133^+^ cells had highest level of invasiveness, knockdown of gal-1 in CD133^+^ cells greatly suppressed their invasive ability to the level comparable to that of CD133^−^ cells. Finally, overexpression of gal-1 (supplemental Fig. S1) in CD133^−^ cells significantly enhanced the invasive capacity, further indicating the critical role of gal-1 on maintaining the phenotypes of cancer stem cells.

### Galectin-1 promotes CD133^+^ lung adenocarcinoma growth *in vivo*

**Figure 5:**
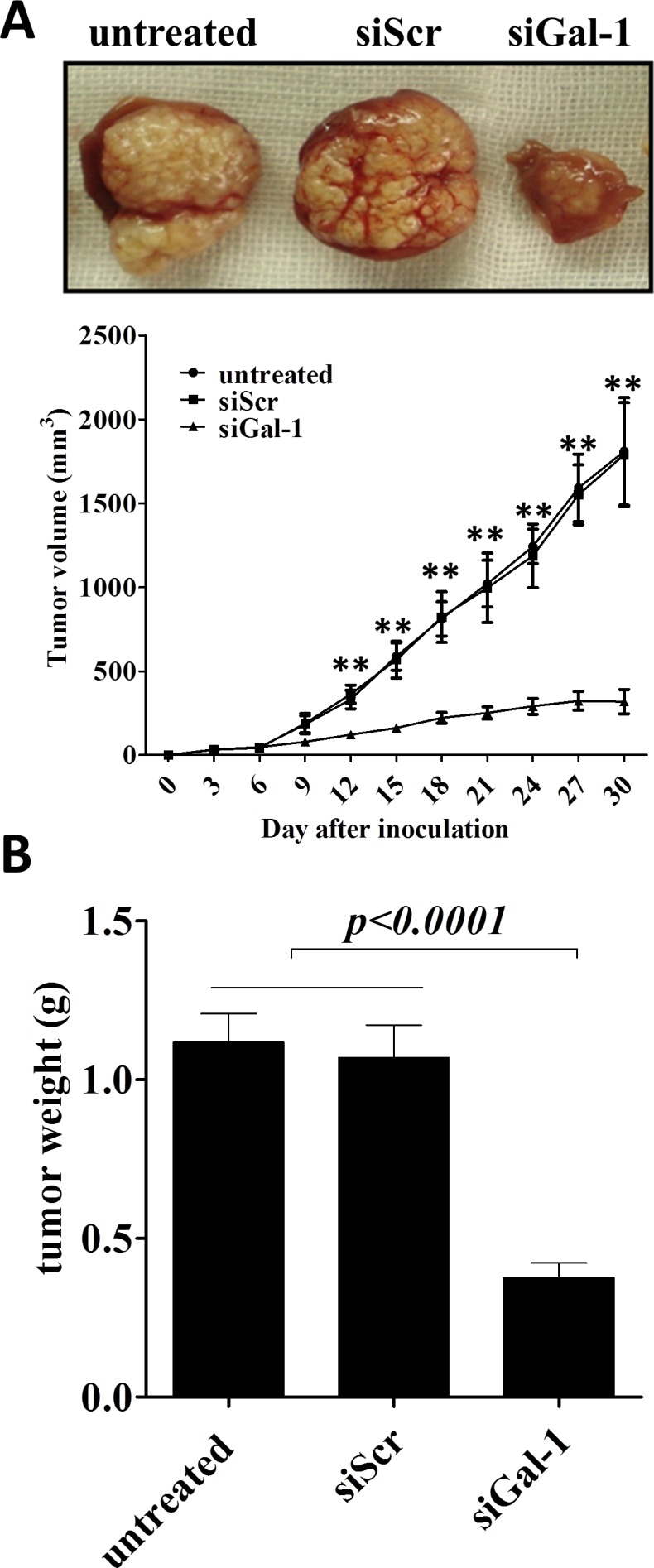
Knockdown of galectin-1 in CD133^+^ cells suppresses the tumor growth in xenograft model in SCID mice (A) SCID mice (n =6 per group) were subcutenously injected with tumor cells (5 × 10^5^ per inject site) on day zero. Tumor volume was monitored every three days, On day 30, the tumors were carefully dissected from the mice and weighed. Data represented as Mean ± SD, n = 3 independent experiments. ** p<0.01.

CD133^+^ lung cancer stem cells can mediate tumorigenesis of lung cancers [28], thus, we further evaluated whether gal-1 could promote CD133^+^ lung adenocarcinoma growth *in vivo*. Wild type (or siScr-transfected) CD133^+^ cancer cells and gal-1 knockdown CD133^+^ cancer cells were injected into the flank of SCID mice, the volume of tumor xenografts were monitored. The results showed that galectin-1 knockdown significantly suppressed growth of xenografts in SCID mice (Fig. 5A) as well as the tumor weights at the end of studies (Fig. 5B). These results suggest that gal-1 is important for *in vivo* tumorigenesis and growth of CD133^+^ lung adenocarcinoma stem cells.

### Suppression of galectin-1 in CD133^+^ lung adenocarcinoma cells results in deactivation of COX2/PGE2 and AKT/mTOR pathways

**Figure 6:**
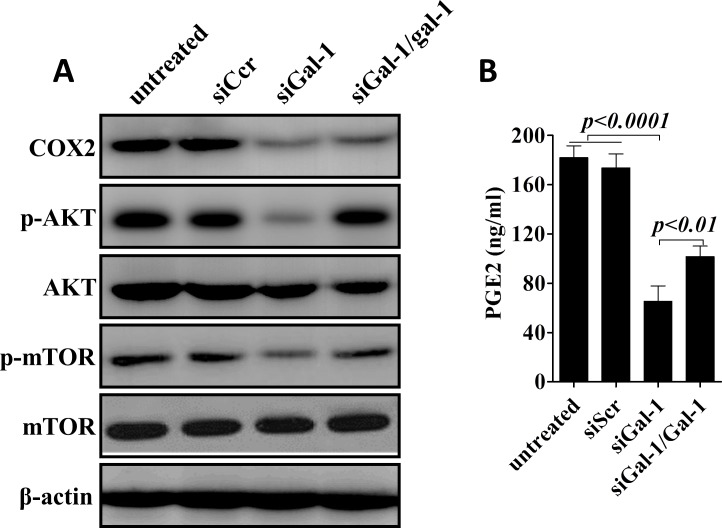
Konckdown of galectin-1 suppresses COX-2/PGE2 and AKT/mTOR signaling pathways (A). Western blot analysis showed expression of COX-2, phosphorylation of AKT and mTOR were greatly reduced after knockdown of gal-1. (B) ELISA revealed that secretion of PGE2 was also inhibited by siGal-1 treatment. Interestingly, when we added recombinant human galectin-1 (rhGal-1; 100 ng/mL) to the culture media for one hour later, treatment of rhGal-1 could partially rescue the effects of siGal-1 on COX-2/PGE2 and AKT/mTOR pathways. Data presented as Mean ± SD, n = 3 independent experiments.

CD133^+^ cells in NSCLC has been found to serves as highly tumorigenic cells (cancer stem cells) and is associated with the maintenance, metastasis and drug-resistance of lung cancers [29, 30]. To examine the role mechanisms underlying gal-1 mediated proliferation, colony formation and invasion in CD133^+^ cells, in this subpopulation cells, gal-1 expression was knockdown in CD133^+^ cells derived from lung carcinoma cells from No. 5 patient in our patient group. Compared with tumor cells transfected with siScr, Since aberrant excessive activation of COX-2/PGE2 and AKT/mTOR pathways are crucial for tumor cell growth survival, and invasion in lung cancers [31-34]. We tested the role of gal-1 on these pathways. We found that gal-1 knockdown led to significantly decreased expression of COX-2, and activation of AKT and mTOR pathways, as well as suppressed secretion of PGE2 in CD133^+^ cells. Interestingly, when we treated the cells with human recombinant gal-1 (100 ng/mL), we can at least partially rescue the effects of knockdown of endogenous gal-1 expression (Fig. 6). Thus, our studies suggested that *first*, gal-1 can function both intracellularly and extracellularly, and *second*, gal-1 might regulate the phenotypes of cancer stem cells through activation of COX-2/PGE2 and Akt/mTOR pathways.

### Increased level of galectin-1 in blood circulation correlates with poor prognosis and high lymph node metastasis of the patients with lung adenocarcinoma

**Figure 7:**
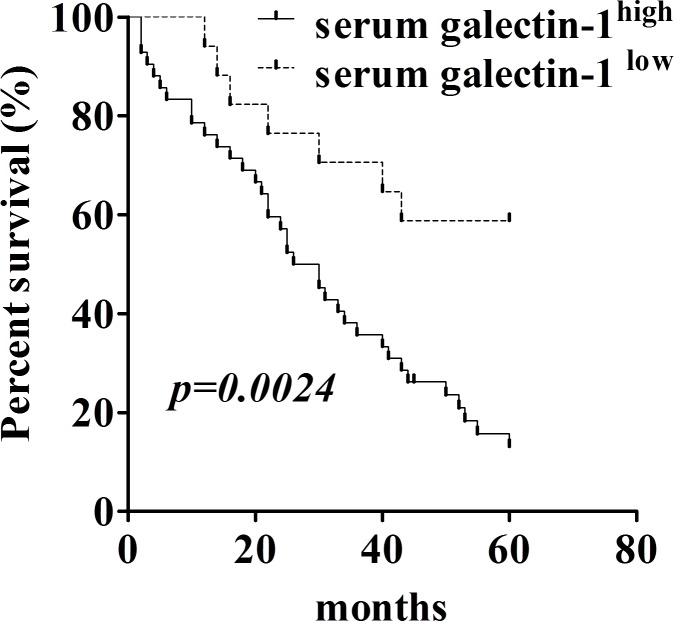
Serum galectin-1 levels correlate with prognosis of the patients with lung carcinoma Kaplan-Meier survival curve revealed overall survival of patients with lung adenocarcinoma (n=66) is significantly correlated with galectin-1 level in blood circulation.

Since our data in Fig. 6 showed extracellular application of gal-1 can regulate the intracellular pathways for tumor growth, our next question was whether the serum level of gal-1 contributes to the progression of lung adenocarcinoma in human patients? If yes, can serum gal-1 serve as a biomarker for prognosis of patients with lung cancers? To answer this question, we analyzed the association between the serum gal-1 concentration and the prognosis of the patients with lung adenocarcinomas by a retrospective cohort study. We separated 66 lung adenocarcinoma patients as two groups (gal-1^high^ and gal-1^low^) according to our established gal-1 measurement protocol. According to our previous studies shown in Fig. 1A, cut off value was set to 49.84 ng/ml, namely the median of serum galectin-1 level of the patients with advanced lung cancers (stage III and IV) subtract 1.96 times of SD, which included 95% of patients in stage III and IV. Five-year survival rate of patients with serum gal-1 level lower than 49.84 ng/ml was 58.824%, while the patients with gal-1 level higher than 49.84 ng/ml was only 13.095%. Kaplan-Meier analysis using the log-rank test revealed significantly negative correlation between gal-1 expression in serum and overall survival rate (Fig. 7, *p=0.0024*). Interestingly, serum level of gal-1 also correlates with lymph node metastasis of the patients, as shown in Table 1, the patients with lymph node metastasis displayed a significantly higher gal-1 level (56.61 ± 14.68 ng/ml) in blood circulation that those without lymph node metastasis (31.54 ± 8.32 ng/ml). Taken together, our data suggested that serum gal-1 level can be useful prognostic marker for the patients with lung adenocarcinoma.

## DISCUSSION

Traditionally, tumors were considered as simply uncontrollable expansion of aberrant cells. However, the growing evidences have led to the emerging concept that the tumors are hierarchically organized and are composed of a heterogeneous population of cells where a rare population called cancer stem cells (CSCs) play a critical role in the development of cancer, especially cancer tumorigenesis [35, 36] and resistance to chemo/rediotherapy [37-39]. Recent studies suggest that specifically targeting CSCs presents therapeutic opportunities to greatly improve the efficacy of tumor therapies [40, 41]. Therefore, the identification and characterization of such population from the rest of cancer cells may contribute significantly to develop novel therapeutic strategies to combat cancers.

In this study, we purified the population of CD133^+^ cells from fresh excised lung adenocarcinoma specimens of 9 patients for our studies. Previous studies have shown gal-1 is a member of the evolutionarily conserved lectin family, and involved in a variety of physiological and pathological processes, including cell adhesion, cell growth regulation, immunomodulation, inflammation, apoptosis, embryogenesis, and cancer progression. Several studies have reported that overexpression of galectin-1 is associated with the proliferation and migration of tumor cells and inhibition of gal-1 inhibition affects tumor-specific immune responses and sensitivity to chemotherapies [21, 42, 43]. In addition, gal-1 was also reported to promote the proliferation of normal stem cells [44, 45], however, to data, there is no study links gal-1 to cancer stem cells.

In the present study, we found that gal-1 predominantly expressed in CD133^+^ lung adenocarcinoma stem cells than CD133^−^ normal cancer cells. This asymmetrical expression pattern of gal-1 in lung adenocarcinoma cells inspired us to further study whether this phenomenon associated with the CSCs mediated tumorigenesis. Indeed, we found that knockdown of gal-1 significantly inhibited proliferation and growth of CD133^+^ cancer cells both *in vitro* and *in vivo*. Colony forming efficiency assay was also showed that the anchoring independent colony formation capacity of CD133^+^ cells was significantly inhibited gal-1 knockdown CD133^+^ cells in comparison with the control cells. Furthermore, a signature of cancer stem cells is the invasive ability, we found that knockdown of gal-1 in CD133^+^ cells significantly suppressed their invasive ability to comparable to that of CD133^−^ cells. Conversely, overexpression of gal-1 in CD133^−^ cells significantly enhanced their invasiveness *in vitro*. Therefore, these results demonstrated that gal-1 plays a vital role in the tumorigenesis of CD133^+^ lung adenocarcinoma cells.

A growing body of evidences indicate that excessive activation of AKT/mTOR and COX-2/PGE2 signal pathways as a common occurrence in human cancers [46, 47], furthermore, inhibitors specific to these pathways has been showed certain antitumor activities, including tumor regressions and prolonged stable disease, which has been reported among patients with a variety of malignancies, including non-small cell lung cancers [48-52]. Like CCI-779 (Temsirolimus) and RAD001 (Everolimus), which have been tested in clinical trials and showed to extend the time of stable disease and the progression-free survival in NSCLC [53]. Gal-1 has been reported to promote stem cells proliferation by up-regulating AKT/mTOR pathway [23, 45], or promote lung cancer progression and chemoresistence by upregulating COX-2/PGE2 pathway [21]. In CD133^+^ lung adenocarcinoma stem cells, we found that gal-1 knockdown significantly reduced COX-2 expression and PGE2 secretion, and the phosphorylation levels of AKT and mTOR, more importantly, exogenous galectin-1 could partially rescue the influence of galectin-1 knockdown. Thus, future experiments are required to study the cellular pathway that controls secretion of gal-1 in the blood stream or the potential function of circulating gal-1 which may represent an additional avenue for treatment of human patients with lung cancers. In addition, the potential differential roles of intracellular and extracellular gal-1 on regulating CSCs should be further studied. For example, since exogenous application of gal-1 can partially rescue the influence of gal-1 knockdown, the further studies should focus on whether exogenous gal-1 can be uptaken by the cells, if yes, which endocytic pathway is involved in this process; if no, is there any receptors for gal-1 on the surface of the cells which can activate downstream pathways.

In summary, combined with our results that high serum levels of galectin-1 showed poor overall prognosis in human patients with lung adenocarcinoma, our studies suggest that gal-1 may be a potential molecular target to treat lung adenocarcinoma and/or a marker for prognosis of the human patients with lung adenocarcinoma.

## MATERIALS AND METHODS

### Human patients

The study was approved by the Ethical Review Board for Research in Wuhan University. A total of 66 patients were given informed consent according to institutional guidelines. The patients were diagnosed with lung adenocarcinoma at Zhongnan Hospital between 2003 and 2013. The clinicopathological parameters of the patients were summarized in Table 1. In addition, serum samples from nine health donors (HD) (from Department of Clinical Laboratory, Zhongnan Hospital) were collected as control. The total of 75 serum samples was measured for the concentration of galectin-1 with established ELISA assay. Serum samples derived from these 66 patients were also used for the retrospective cohort analysis of survival data of five year follow-up.

### Magnetic activated cell sorting

Among the total 66 patients, there were nine patients with advanced lung adenocarcinoma were enrolled for tumor cells collection. Surgically resected adenocarcinoma specimens and 10 ml corresponding peripheral blood were collected. Fresh tumor tissues were obtained by surgical resection and washed 3 times with PBS and left overnight in DMEM–F12 medium (Hyclone) supplemented with penicillin (200 U/ml), amphotericin B (20 μg/ml) and streptomycin (200 μg/ml). Then the tumor tissues were gently disrupted and cells were isolated by density centrifugation as described previously [54-56]. Tissue dissociation was carried out by enzymatic digestion (20 μg/ml collagenase II, Invitrogen) at 37^o^C for 2h. In order to avoid potential contamination from infiltrating blood cells which can also be CD133^+^, such as CD45^+^ CD133^+^ cells, recovery cells were cultured in serum-free DMEM–F12 medium supplemented with 100 μg/ml apo-transferrin, 10 μg/ml putrescine, 2 μM progesterone, 50 μg/ml insulin, 0.03 mM sodium selenite, 0.1% sodium bicarbonate, 0.6% glucose, 5 mM HEPES, 0.4% BSA, glutamine,antibiotics, 20 μg/ml Epidermal Growth Factor (EGF) and 10 μg/ml basic Fibroblast Growth Factor (bFGF). The floating cells (infiltrating blood cells and dead cells) were removed by PBS washing before flow cytometry sorting. Single cells were prepared with enzymatic digestion after 3 subcultures, then the cells were labeled with magnetic anti-CD133 MicroBeads (Miltenyi Biotec, Germany) for 30 min at room temperature and applied to the prepared MS Column (Miltenyi Biotec, Germany). CD133^+^ and CD133^−^ cells were collected respectively for future experiments.

### Flow cytometry

The percentages of CD133^+^ cells in the original cell populations and the two isolated cell populations were analyzed by flow cytometry. Briefly, after three times washing with PBS, cells (1×10^6^) were incubated with a PE-conjugated anti-CD133 antibody or isotype control antibody (Miltenyi Biotec, Germany). After 30 min incubation at 4 ^o^C, cells were washed for three times and analyzed with flow cytometer (Beckman coulter).

### RNA isolation and real-time reverse transcription polymerase chain reaction

Total RNA was extracted from the cells using TRIZOL (Invitrogen). Gal-1 mRNA was quantitated by the real-time RT-PCR using the QuantiTect ^TM^ SYBR Green PCR Handbook Kit (QIAGEN). The oligonucleotide primers were as follows, galectin-1 forward: 5′-TACTGAAGGCCGTGGAC-3′, reverse: 5′-CCTCGTGGCTCCA GTACA-3′; β-actin forward: 5′-TACTGAAGGCCGTGGAC-3′, reverse: 5′-CCTCGTGGCTCCA GTACA-3′. The PCR protocol was carried out as the following: the samples were heated to 95^o^C for 10 min, followed by 30 cycles of 95^o^C for 10 secs, 53 ^o^C for 15 secs, and 72 ^o^C for 20 secs. Then the final extension was carried out by incubation at 72 ^o^C for 10 mins. The relative expression of galectin-1 was normalized by β-actin as an internal control.

### Western blotting

For Western blotting analysis, the cells were lysed on ice for 30 min in a lysis buffer containing fresh protease and phosphotase inhibitor cocktails (Sigma). The lysates were then centrifuged (12000 g at 4 °C for 15 min). Protein concentrations were determined by protein assay (Bio-Rad, Hercules, CA). Equivalent proteins were denatured in protein loading buffer, loaded onto 10% SDS-PAGE gels, and subsequently transferred to polyvinylidene difluoride (PVDF) membranes (Millipore, Billerica, MA) by electroblotting. The PVDF membranes were blotted with 5% nonfat milk in TBST buffer (Bio-Rad) for 1 h and incubated overnight at 4°C with antibodies against galectin-1, COX2, β-actin (Abcam) and AKT, p-AKT, mTOR, p-mTOR (CST). Signals were detected using ECL detection reagents (Perkin-Elmer Life Sciences, Boston, USA) following the manufacturer's instructions.

### Galectin-1 Interference

The sense sequence of the galectin-1 siRNA (siGal-1) used in the current work was 5′-GCUGCCAGAUGGAUACGA ATTTT-3′ and the antisense sequence was 5′-UUCGUAUCCAUCUGGCAGCTTTT-3′. The sense sequence of the scramble siRNA (siScr) was used as a control (sense: 5′-CUACGAUGCUGCUUAGCUCTTTT-3′ and antisense: 5′-GAGCUAAGCAG CAUCGUAGTTTT-3′) [57]. The siGal-1 and siScr control we used in the current study have been proven to be the most efficient among several siRNAs against gal-1 in a previous study [58]. Then these two pairs of siRNA were subcloned into pSilencer 3.1-H1 neo expression vector (Ambion, Austin, TX) between BamHI and HindIII sites to construct pSilencer 3.1-H1/siGal-1-producing siRNA targeting gal-1 or a scramble control. CD133^+^ tumor cells were transfected with these two constructed vectors using Lipofectamine 2000 reagent (Invitrogen, Carlsbad, CA) according to the manufacturer's instructions. Stable clones were generated by culturing the cells with G418 (600 μg/ml) in culture medium (Invitrogen).

### Enzyme linked immunosorbent assay

The gal-1 level in serum (10-fold dilution) or culture media (2-fold dilution) were determined by Human Galectin-1 Quantikine ELISA Kit (R&D Systems), PGE2 concentration in cell culture media was determined by Prostaglandin E2 ELISA Kit (abcam) according to the protocol recommended by manufacturers.

### Cell proliferation assay

MTT assay was used to determine the proliferation of the cells with different treatments. Briefly, cells were suspended and diluted to 1.25×10^4^ cells/ml in fresh medium, and seeded into 96-well plates (100 μl/well). The plates with cells were cultured and one plate was used for MTT assay each day for a total of 4 days. For measurement, 20 μl of 3-(4,5-Dimethylthiazol-2-yl)-2,5-Diphenyltetrazolium Bromide (MTT) (5 mg/ml) was added to each well, and the cells were incubated at 37 ^o^C for another 4 h. Then the culture medium was replaced with 150 μl dimethyl sulfoxide (DMSO, Amresco, USA). After incubating the plates on a shaker for 10 min, the absorbance was detected at 590 nm on a plate reader. The proliferation rate was determined as follows: absorbance of transfected cells / absorbance of untransfected cells.

### Invasion assay

Transwell inserts (8 μm pore; Corning Costar) were placed into the six-well plate. BD Matrigel (BD Biosciences) was hydrated with 10% FBS-DMEM. A total of 5 × 10^4^ cells in 0.5% FBS-DMEM were placed in the upper chamber and 10% FBS-DMEM were added in the lower chamber. After 48 h incubation, the cells that had invaded another side of the membrane were fixed with methanol for 15min and then stained with crystal violet for 20 min. the numbers of invasive cells were counted under microscope.

### Anchorage-independent growth assay

One and half ml of 0.5 % agar supplemented with RPMI, 10 % FBS, were plated in six-well plates as bottom agar. One thousand cells were mixed with 1.5 ml of 0.35 % agar supplemented with RPMI, 10 % FBS, and plated on the solidified bottom agar. One ml of media was added on top of the solidified agar layers and the colonies were allowed to grow for 14 to 20 days. The images of cell colonies were captured with an inverted microscope [59].

### Xenograft mouse model

Six-to-eight week's old female SCID mice were purchased from Laboratory Animal Center of Wuhan University (Wuhan, China) and maintained in the cages under sterile conditions with a specific pathogen-free environment. Mice were injected subcutaneously with siRNAs transfected or untransfected cells (5×10^5^) in a volume of 100 μl in the flank at the time of inoculation. Tumor size was measured in perpendicular dimensions every three days, and tumor volume was calculated as 1/2 × (tumor length) × (tumor width)^2^. The mice were sacrificed and the tumors were dissected and weighted on the 30^th^ day after inoculation.

### Statistical analysis

Data presented as the Mean ± SD and analyzed by Graphpad Prism V.5.00 software (GraphPad Software, San Diego CA, USA). Differences among different groups were tested by one-way ANOVA followed by Neuman-Keuls post hoc test. Overall patient survival was calculated from the date of diagnosis to the date of last follow-up (censored) or the date of patient death by any cause (event). Survival probabilities were calculated using the Kaplan-Meier method. Differences in survival between patient subgroups were analyzed using log-rank (Mantel-Cox) Test. Spearman rank test was performed to show a positive correlation between cancer stage and serum galectin-1 level. A two-side *p* values under 0.05 were considered statistically significant.

## SUPPLEMENTARY MATERIAL AND FIGURE


