# Supplementary material for: Galectin-1 is overexpressed in CD133+ human lung adenocarcinoma cells and promotes their growth and invasiveness
Source: Oncotarget. 2014 Dec 26;6(5):3111–22. doi: 10.18632/oncotarget.3076 (PMC4413641; doi:10.18632/oncotarget.3076)
Supplement: Supplementary file 1 [file oncotarget-06-3111-s001.pdf]

Galectin-1 is overexpressed in $CD133^{+}$ human lung adenocarcinoma cells and promotes their growth and invasiveness

Supplementary Material

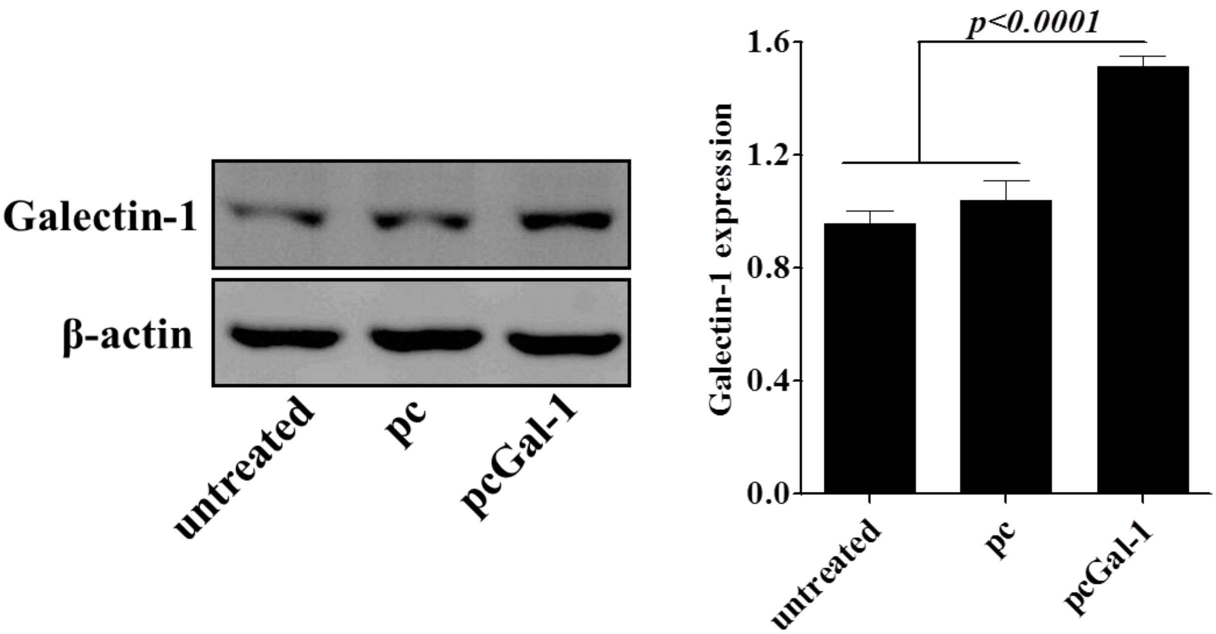

Supplemental figure S1: Western blot analysis shows overexpression of gal-1 in $CD133^{-}$ cells. $CD133^{-}$ cells were transfected with either plasmid containing gal-1 cDNA and empty plasmid as a control. Twenty four hours later, the cells were harvested for western blot analysis. The density of each band was quantified by ImageJ software (NIH). Data presented data showed as Mean ± SD, n = 3 independent experiments.
